# Modernizing the Staging of Parkinson Disease Using Digital Health Technology

**DOI:** 10.2196/63105

**Published:** 2025-04-04

**Authors:** John Michael Templeton, Christian Poellabauer, Sandra Schneider, Morteza Rahimi, Taofeek Braimoh, Fhaheem Tadamarry, Jason Margolesky, Shanna Burke, Zeina Al Masry

**Affiliations:** 1 Bellini College of Artificial Intelligence, Cybersecurity, and Computing University of South Florida Tampa, FL United States; 2 Department of Medical Engineering University of South Florida Tampa United States; 3 Knight Foundation School of Computing and Information Sciences Florida International University Miami, FL United States; 4 Department of Communicative Sciences and Disorders Saint Mary's College Notre Dame, IN United States; 5 Department of Neurology Miller School of Medicine University of Miami Miami, FL United States; 6 Robert Stempel College of Public Health & Social Work Florida International University Miami, FL United States; 7 FEMTO-ST Institute Ecole Nationale Supérieure de Mécanique et des Mécatroniques Besançon France

**Keywords:** digital health, Parkinson disease, disease classification, wearables, personalized medicine, neurocognition, artificial intelligence, AI

## Abstract

Due to the complicated nature of Parkinson disease (PD), a number of subjective considerations (eg, staging schemes, clinical assessment tools, or questionnaires) on how best to assess clinical deficits and monitor clinical progression have been published; however, none of these considerations include a comprehensive, objective assessment of all functional areas of neurocognition affected by PD (eg, motor, memory, speech, language, executive function, autonomic function, sensory function, behavior, and sleep). This paper highlights the increasing use of digital health technology (eg, smartphones, tablets, and wearable devices) for the classification, staging, and monitoring of PD. Furthermore, this Viewpoint proposes a foundation for a new staging schema that builds from multiple clinically implemented scales (eg, Hoehn and Yahr Scale and Berg Balance Scale) for ease and homogeneity, while also implementing digital health technology to expand current staging protocols. This proposed staging system foundation aims to provide an objective, symptom-specific assessment of all functional areas of neurocognition via inherent device capabilities (eg, device sensors and human-device interactions). As individuals with PD may manifest different symptoms at different times across the spectrum of neurocognition, the modernization of assessments that include objective, symptom-specific monitoring is imperative for providing personalized medicine and maintaining individual quality of life.

## Introduction

Neurodegenerative disorders are now the leading cause of disability in the world, the fastest growing being Parkinson disease (PD), which it is estimated will impact 14.2 million people worldwide by the year 2040 [1]. PD is often described as a “designer disease, ” meaning that individuals with PD manifest different neurocognitive symptoms (ie, motor, memory, speech, language, executive function, autonomic function, sensory function, behavior, and sleep problems [2]) at different times and to a variable extent across the spectrum of neurocognition and each individual’s course of their disease [3]. Due to this variability in presentation, an early and accurate diagnosis of PD can be difficult [4,5]. Given this difficulty, every effort must be made to improve diagnostic accuracy in PD [6] to provide individuals with PD with more accurate prognostic information, facilitate enrollment in early disease-modifying trials in PD, and initiate therapeutics to improve quality of life.

Fortunately, the pervasiveness of digital health technology (eg, smartphones, tablets, and wearable devices) and the substantial increase in its use in clinical settings (ie, with a more than 5-fold increase over the past 5 years and an expected 10-fold growth in the next 3 to 5 years [7]) provides a prudent opportunity to address this problem by implementing (1) innovative sensing modalities (eg, device sensors and human-device interactions [8]), (2) accurate PD detection through advanced computer-assisted techniques (eg, artificial intelligence [AI] [9] and machine learning [ML] [10]), and (3) continuous monitoring of PD in daily life across all functional areas of neurocognition [11]. To deepen the understanding between digital health technology (eg, for the collection and analysis of objective sensor-based features) and the classification of PD and its stages, we provide an inspired viewpoint at the nexus of these 2 research topics. This paper is intended to identify both promising approaches and gaps in the current research for clinicians and researchers. Finally, this viewpoint proposes the foundation for a new staging schema that addresses these promising approaches and gaps via the implementation of digital health technology in an effort to modernize current staging protocols such that they encompass an objective assessment of all functional areas of neurocognition.

## Related Work

### Clinical Assessment Techniques

Due to the complicated nature of PD, a number of subjective considerations on how best to assess clinical deficits have been published [12]. There are currently multiple staging schemes, clinical assessment tools (eg, screening assessments and functional movement assessments), and questionnaires used to assess all necessary neurocognitive functions of interest as presented in Table 1.

**Table 1:** Current assessments of Parkinson disease across all neurocognitive functions of interest.

Functions	Assessments															
	Staging scales				Screening			Comprehensive			Functional movement assessments					Patient-reported outcomes
	H&Y^a^	MDS-UPDRS^b^	BSS^c^	MMSE^d^		MoCA^e^	NFI^f^		DWNAS^g^	BBS^h^		STS^i^ test	TUG^j^ test	6MWT^k^	PDQ-39^l^	
Motor	✓^m^	✓		✓		✓	✓		✓	✓		✓	✓	✓	✓	
Memory				✓		✓	✓		✓						✓	
Speech		✓		✓		✓	✓		✓						✓	
Language				✓		✓	✓		✓						✓	
Executive				✓		✓	✓		✓						✓	
Sensory									✓						✓	
Behavioral		✓					✓		✓						✓	
Sleep		✓													✓	
Autonomic		✓													✓	

^a^H&Y: Hoehn and Yahr Scale [13].

^b^MDS-UPDRS: Movement Disorder Society–Unified Parkinson’s Disease Rating Scale [14].

^c^BSS: Braak staging scheme [15].

^d^MMSE: Mini-Mental State Examination [16].

^e^MoCA: Montreal Cognitive Assessment [17].

^f^NFI: Neurobehavioral Functioning Inventory [18].

^g^DWNAS: Dean-Woodcock Neuropsychological Assessment System [19].

^h^BBS: Berg Balance Scale [20].

^i^STS: sit-to-stand [21].

^j^TUG: timed up-and-go [22].

^k^6MWT: 6-minute walk test [23].

^l^PDQ-39: Parkinson’s Disease Questionnaire-39 [24].

^m^The corresponding staging scale or assessment evaluates the associated function.

Staging methods, such as the Hoehn and Yahr Scale (H&Y) [13] (an observational classification method based on accepted cardinal motor signs: “rest” tremor, rigidity, bradykinesia, and impaired postural and righting reflexes) and the Movement Disorder Society–Unified Parkinson’s Disease Rating Scale (MDS-UPDRS) [14] (a scale developed to incorporate elements from existing scales to provide an efficient, flexible, and comprehensive means to monitor motor and self-reported nonmotor PD symptoms), are commonly used to depict the progression of PD. Other methods such as the Braak staging scheme [15] (a neurobiological pathology approach to staging PD) have been developed, although they are not commonly used clinically. Assessment methods include both screening and comprehensive tools. Screening assessment tools, which include the Mini-Mental State Examination [16] and the Montreal Cognitive Assessment [17], provide a quick general assessment of an individual with suspected neurocognitive impairment and identify areas needing further comprehensive evaluation. These assessments focus on a range of neurocognitive functions. More comprehensive assessments such as the Neurobehavioral Functioning Inventory [18] and Dean-Woodcock Neuropsychological Assessment System [19] aim to assess additional neurocognitive areas of interest or provide a more in-depth assessment. Functional movement assessments, including the Berg Balance Scale [20], timed up-and-go test [22], sit-to-stand test [21], and 6-minute walk test [23], are all commonly used by clinicians (eg, physical therapists and occupational therapists) to assess functional motor performance. Finally, patient-reported outcomes (PROs) come from specific questionnaires (eg, Parkinson’s Disease Questionnaire-39) used in the routine monitoring of PD in which the individual assesses their mobility, activities of daily living, emotional well-being, stigma, social support, cognition, communication, and bodily discomfort [24].

However, none of these staging schemes, clinical assessment tools, or questionnaires include an objective assessment of all functional areas of neurocognition affected by PD, and there is a noted lack of consistency. All listed progression scales and assessment tools differ in a multitude of ways, including the type of classification being completed (eg, observational vs pathological), the number of functional areas of neurocognition assessed (eg, motor, memory, speech, language, executive function, autonomic function, sensory function, behavior, and sleep), the total number of classified stages in the scale being used, and importantly the subjective manner (eg, via interrater variability and variable performance of the practitioner) in which the information for each individual was gathered and assessed.

### Digital Health Technology

As the prevalence of digital health technology (eg, smartphones, tablets, and wearable devices) and the subsequent collection of large amounts of complex health data increase, mobile devices provide clinicians (eg, epidemiologists, neurologists, physicians, and other health care personnel) with a robust way to collect, analyze, and interpret new digital biomarkers related to neurodegenerative diseases [25,26]. Mobile device capabilities allow for the expansion of clinically relevant functional assessments and the collection of objective symptom-specific information (eg, digital biomarkers) through the use of device-based sensors (eg, accelerometers, gyroscopes, GPS, microphones, cameras, and timers) and human-device interactions (eg, screen interactions) [2,27-30]. Mobile devices can also use opportunistic approaches to monitoring (eg, having device sensors on in the background), which allows for the collection of additional objective features [2]. In addition, standardized health screenings, clinician observations, and PROs can be collected via mobile devices to be used for individual evaluation [31]. This combination of digital biomarkers, clinician observations, and PROs allows for the collection of relevant health information and monitoring of all functional areas of neurocognition (eg, motor, memory, speech, language, executive function, autonomic function, sensory function, behavior, and sleep) [2]. With the increased volume of relevant health data, novel ways to interact with and extract meaning from the data emerge [26,32]. ML is a key technique that has demonstrated the ability to translate these large health datasets into actionable knowledge [33-35]. Specifically, supervised ML and AI-enabled detection of health data has shown potential in the area of disease prediction, classification, and monitoring of overall progression [36-39]. Furthermore, with the increased opportunity for users’ participation on their own devices and the ability of the clinician to collect and analyze enhanced objective datasets, the continued development and integration of mobile health technologies into the routine assessment and care of patients with PD can allow for more sophisticated characterization of patients’ function, better tailoring of symptomatic therapy, greater patient engagement and self-assessment, and overall improved health care outcomes [40].

### Modernizing the Staging of PD

**Figure 1:**
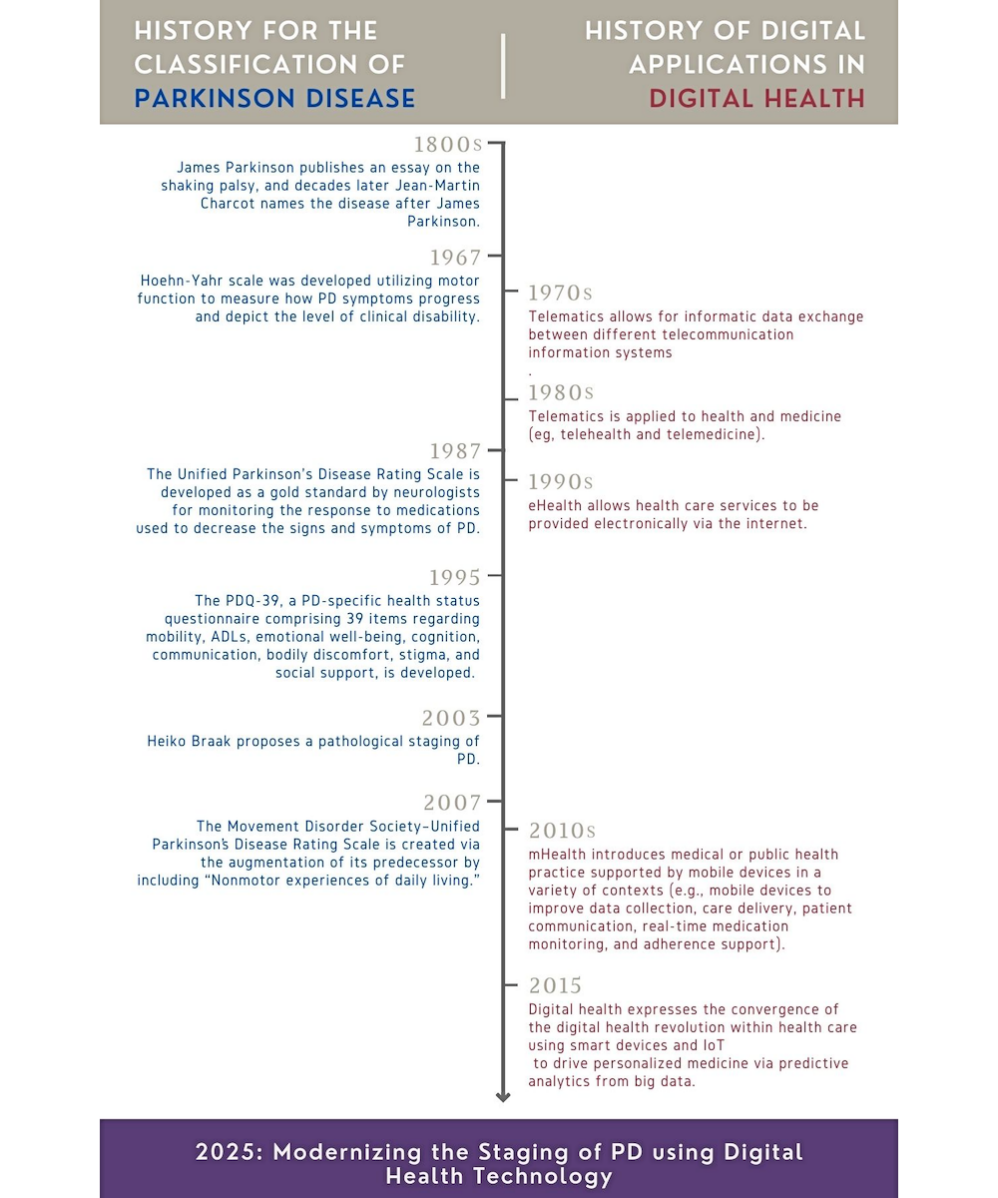
Timeline of both the history of the classification of Parkinson disease (PD) and the history of digital health technology. ADL: activity of daily living; IoT: Internet of Things; mHealth: mobile health; PDQ-39: Parkinson’s Disease Questionnaire-39.

Given the crossover nature of this viewpoint, Figure 1 was created to present a timeline of the history of the classification of PD alongside the history of digital health technology. In the past 60 years, there has been a tangential modernization of both classification and staging techniques and digital health technology. To further preface the viewpoint expressed herein, Figure 1 also fosters the premise that additional PD classification developments should be explored, given the many convergent advances in the field of digital health technology as well as the expected growth of mobile-based health therapeutics in the next 3 to 5 years (7).

## Viewpoint Basis

To understand the highly diverse use of digital health technology for the assessment and monitoring of PD, an extension of the Related Work subsection is provided to set a foundation of insights on the current state of technology, strategies of use, and future opportunities. We acknowledge that the past 4 to 5 years are more relevant and interesting in terms of using novel digital health technology for the assessment of PD; however, studies from 2015 onward were reviewed to allow for the depiction of efforts using digital health technology for the assessment of PD (Figure 1).

As this extension of the Related Work subsection explores the nexus of PD and digital health technology (eg, smartphones, tablets, and wearable devices) specifically related to the assessment of both motor and nonmotor functions associated with PD, this extended literature search used both PubMed and IEEE Xplore databases due to their status as premier resources in the fields of health and engineering, respectively. Specific search queries incorporated the following keywords or terms in both databases: (“Parkinson disease” AND “digital”) OR (“Parkinson disease” AND “mobile”). Search results were limited to English-language full-text articles published between 2015 and 2024. This time range was chosen to enable us to focus on high-quality, state-of-the-art research studies at the nexus of the digital revolution and health care (eg, using digital devices and the Internet of Things to drive personalized medicine via predictive analytics from big data).

Initially, 193 articles were collected from these databases. After reviewing the titles and abstracts of the articles, 74 (38.3%) of the 193 articles were excluded due to the reasons presented in Textbox 1. Of the included 119 articles, Table 2 gives a breakdown by article focus (eg, articles addressing only nonmotor functions, articles addressing only motor functions, and articles addressing both motor and nonmotor functions associated with PD).

**Exclusion criteria used for literature selection.**

**Exclusion criteria**

- Articles that are book chapters, magazines, company reports, white papers, or abstracts
- Studies wherein the digital or mobile focus is related to biological assays or medical imaging analysis (eg, manuscripts that strictly regard data analysis from sources other than smartphones, tablets, or wearable devices)
- Studies wherein the digital or mobile focus is related to the usability of mobile devices for intended populations (eg, manuscripts that strictly regard how a member of the affected population should use a device)
- Studies wherein the focus is related to the creation of digital or mobile stimulation tools to help treat movement disorders (eg, manuscripts that strictly regard a mobile stimulation treatment protocol)
- Studies wherein the focus is related to the formation of digital or mobile adherence tools to help treat movement disorders (eg, manuscripts that strictly regard using mobile devices to log treatment participation)
- Studies wherein the term mobile is solely related to mobility in exercise
- Duplicate articles

**Table 2:** Breakdown of systematic review results by article focus (n=119).

Focus	Articles, n (%)
Multiple neurocognitive (ie, motor and nonmotor) functions	50 (43.1)
Only motor functions	45 (37.8)
Only nonmotor functions	24 (20.2)

**Figure 2:**
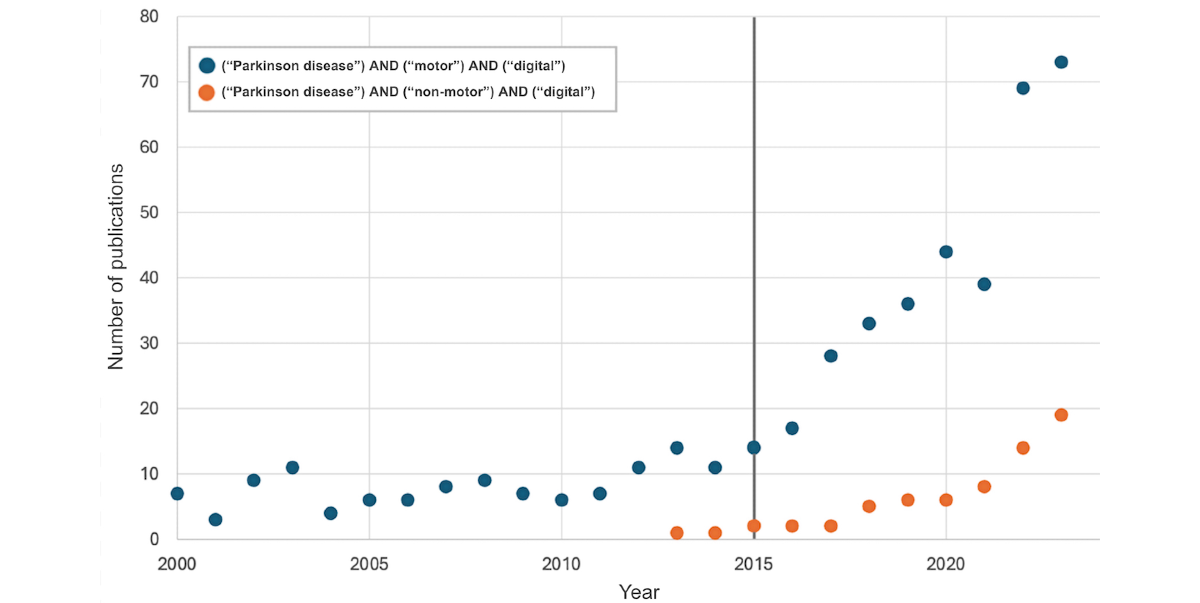
Chronological depiction of publications on digital health technology for the assessment of both motor and nonmotor symptoms of Parkinson disease (PD), highlighting the emergence of “digital health” from 2015 to the present.

While key highlights of this historical crossover are presented in Figure 1, specific points that emphasize and advocate for the use of digital health technology (eg, smartphones, tablets, and wearable devices) for the modernization of the assessment, classification, staging, and longitudinal monitoring of PD are presented in Tables S1-S3 in Multimedia Appendix 1 [4,7,8,11,25,40-115]. These key assertions propel the viewpoint that digital health technology can and should become a prominent driver in the modernization of clinical PD staging protocols. Furthermore, the literature findings in Table 2 highlight that >62% (74/119, 62.2%) of the articles have a focus on nonmotor functionality being assessed using digital health technology, which expresses the need for current staging protocols to be expanded and inclusive of all functional areas of neurocognition (eg, motor, memory, speech, language, executive function, autonomic function, sensory function, behavior, and sleep [2]). Similarly, since the digital health revolution in 2015, as previously noted, there has been a notable increase in publications addressing the prevalence of the use of digital health technology for assessing both motor and nonmotor symptoms for PD (Figure 2). Finally, Table 3 presents a reference-based depiction of the extended literature review findings with a focus on various device sensors and human-computer interactions for the objective assessment of each functional area of neurocognition (eg, motor, memory, speech, language, executive function, autonomic function, sensory function, behavior, and sleep) as well as additional auxiliary information.

Although Figure 2 and Tables 2 and 3 present a large concentration of prior work in the assessment of motor-based symptoms using digital devices, it is expressly noted that there is a prominent shift into the quantitative assessment and analysis of nonmotor symptoms for individuals with PD that are not yet included in currently administered staging schemes (eg, MDS-UPDRS).

**Table 3:** Cross-referenced list of device sensors and capabilities that allow for feature collection relating to each neurocognitive function in addition to important auxiliary information.

Sensors or HCI^a^		Functional areas of neurocognition										Auxiliary information		
		Motor	Memory	Speech	Language	Executive	Sensory	Behavioral	Sleep	Autonomic	Activity		Social	Treatment
**Devices: smartphone and tablet**														
	Accelerometer and gyroscope	[11, 41-45, 116- 125]	[42]	—^b^	—	[42]	—	[42]	[42]	—	[11, 41-45, 116- 125]		—	—
	GPS	[41,117]	—	—	—	—	—	—	—	—	[41,125]		—	—
	Microphone	—	—	[4,42, 43,116, 118, 123, 126- 131]	—	[116]	—	[11,41]	—	—	—		—	—
	Speaker	—	—	—	—	—	[42,46]	—	—	—	—		—	—
	Camera	[42,47, 132]	—	[48]	—	—	—	—	—	—	—		—	—
	Timer	[43, 129]	[4, 133]	[48, 127, 128]	—	[133]	[46]	—	—	—	—		—	—
	Touch interface	[41,49, 116, 118, 121, 123, 130, 134, 135]	[123, 133]	—	—	[121, 133]	—	[7]	—	—	—		—	—
	Pressure	[11,42, 117, 123, 129]	—	—	—	[11]	[46]	—	—	—	—		—	—
	App use	[42-44, 50,57, 119, 122, 124, 136, 137]	[45,50, 116,119, 130,136, 138]	[43,50, 119, 130]	[43,50, 130]	[45,50, 130]	—	[50,51, 130]	[7,50, 139]	—	[50, 117, 139]		[50, 119]	—
	Device use	[42, 138, 140]	—	—	—	—	—	[140]	—	—	[140]		—	—
**Devices: wearables**														
	Accelerometer and gyroscope	[11,42, 43,50, 52-54, 116- 119, 122- 125, 137, 139, 141- 145]	—	—	—	—	—	—	[42, 50, 117, 122, 125, 141, 146]	—	[42]		—	—
	GPS	[141]	—	—	—	—	—	—	—	—	[141]		—	—
	Microphone	—	—	[25]	—	—	—	—	—	—	—		—	—
	Touch interface	—	—	—	—	—	—	—	—	—	—		—	[43]
	PPG^c^	[42]	—	—	—	—	—	[117]	[42]	[50,117, 124,141]	[50]		[50]	—
	EDA^d^	[42]	—	—	—	—	—	[50]	—	[50,131,141]	—		—	—
**Digital survey**														
	Personal information	—	—	—	—	—	—	—	—	[55]	[55]		[50,55]	—
	Clinical information	[42,50, 55,56, 117, 119, 138, 147]	[42,55, 56, 130, 147]	[55,56, 130, 147]	[55,56, 130, 147]	[55,56, 130, 147]	[55,123]	[55,119]	[55, 117]	[55,119, 123]	[50, 55, 117]		[55]	[55,56, 119, 139]
	Quality of life	[45,50, 56, 123, 138, 140, 147]	[41,42, 56, 119, 131, 138, 147]	[41,42, 45,56, 119, 131, 138, 147]	[41,42, 56, 119, 131, 138, 147]	[41,42, 45,56, 119, 131, 138, 147]	[41,42, 50,119, 131, 138, 147]	[41,42,45, 50,56,119, 123, 131, 138, 140, 147]	[41, 42, 50, 117, 119, 123, 131, 138, 147]	[41,42,50, 119,131, 138,147]	[42,45, 50,55, 56,119, 123, 138, 140, 147]		[42,45, 50, 55, 56,119, 123, 138, 147]	—
	Caretaker survey	[147]	[147]	[147]	[147]	[147]	[50,147]	[147]	[147]	[147]	[50,138, 147]		[138, 147]	—

^a^HCI: human-computer interaction.

^b^Not applicable.

^c^PPG: photoplethysmography.

^d^EDA: electrodermal activity.

## Proposed Schema

Given the gaps in current staging schemes (ie, the inability to provide an objective assessment of all functional areas of neurocognition affected by PD), the use of mobile devices (eg, for the collection and analysis of objective digital biomarkers across all functional areas of neurocognition) is available and imperative for the augmentation of traditional assessments into a comprehensive, symptom-specific, and longitudinal approach to the monitoring of PD [40]. Proposed methods for the integration of mobile devices include regularly scheduled clinical assessments, telemedicine-based interactions (eg, for supplemental clinical visits), opportunistic collections (eg, to gather ample, fine-grained health data with little to no interaction from the user), intervention protocol assessments (eg, to understand the short- and long-term benefits of different pharmacological cycles and intervention therapies), and ML methods (eg, for disease prediction, classification, and intervention recommendation). Furthermore, ML can be used for the creation of a novel, comprehensive staging protocol encompassing all areas of neurocognition. This protocol would emerge through the analysis and interpretation of the patterns found in objective symptom-specific features, PROs, and clinician observations, all of which can be collected using mobile devices. These mobile device capabilities ultimately allow for the collection of far more data over time (eg, compared to biannual or annual clinical visits) and provide more accurate measures using device sensors, in addition to reducing bias and interrater variability [117]).

**Figure 3:**
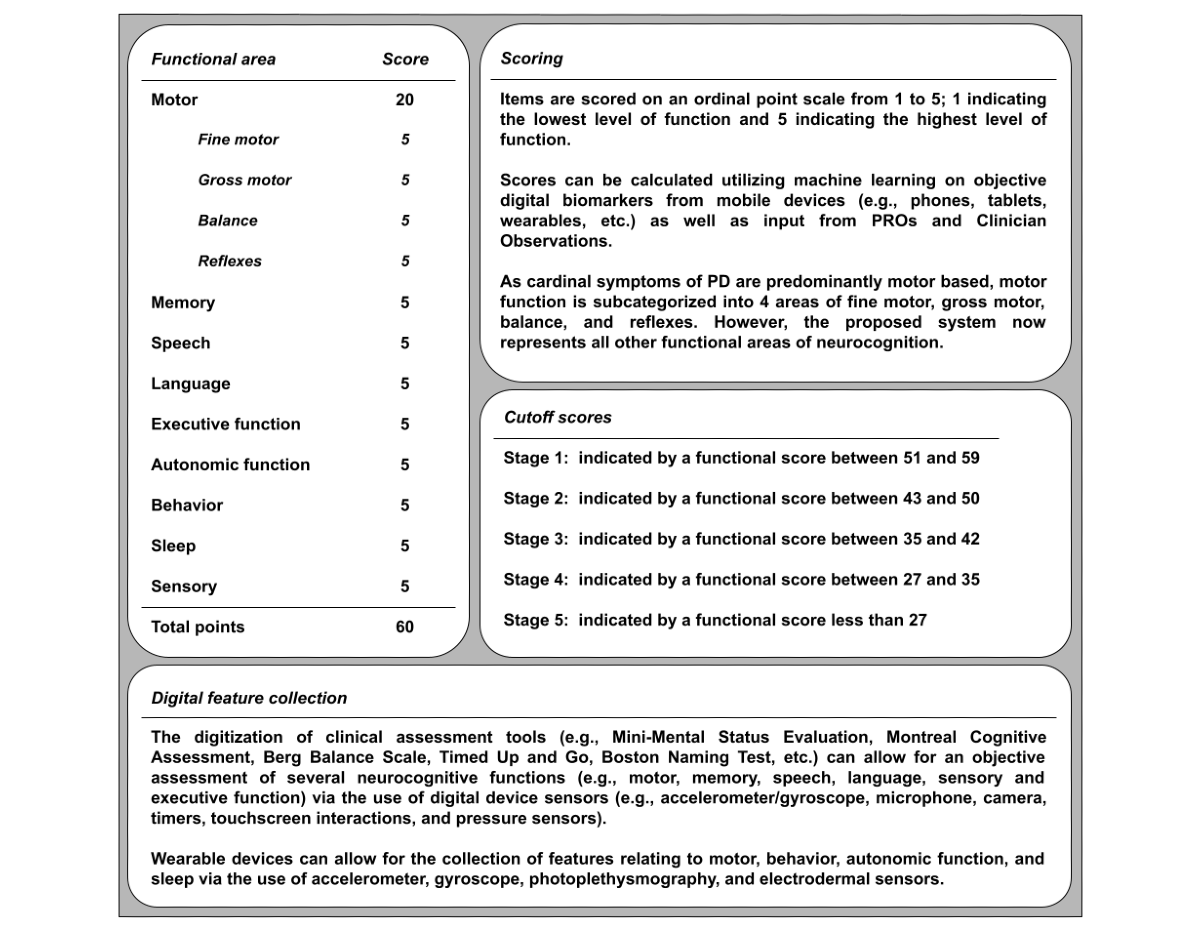
A proposed foundation for a staging schema that builds on the structure of clinically implemented scales (eg, Hoehn and Yahoo Scale, Movement Disorder Society–Unified Parkinsons Disease Rating Scale and Berg Balance Scale) that emphasize motor functionality—fine motor, gross motor, balance, and reflexes—while also integrating all other functional areas of neurocognition related to Parkinson disease (PD; eg, memory, speech, language, executive function, autonomic function, behavior, sleep, and sensory function). This schema would allow for a nuanced, weighted approach to tracking PD progression while also enabling greater insights into the possible subtyping of PD through the use of digitized assessment tools for the collection of objective data. PRO: patient-reported outcome.

Therefore, a proposed foundation for a novel staging schema, encompassing all functional areas of neurocognition, is presented in Figure 3. The foundation of this proposed staging schema builds from multiple clinically implemented scales (eg, H&Y [13], MDS-UPDRS [14], and Berg Balance Scale [20]) for ease and homogeneity while also including objective, symptom-specific assessments of each functional area of neurocognition via inherent device capabilities (eg, device sensors and human-device interactions).

As the cardinal symptoms of PD are motor in nature, predominant staging methods (eg, H&Y) use motor function to measure how PD symptoms progress and depict the level of clinical disability. The H&Y stages PD as follows:

1. Symptoms are present on 1 side only (unilateral).
2. Symptoms are present on both sides, but there is no impairment of balance.
3. There are balance impairments (eg, anterior or posterior postural instability as assessed by the pull test) as well as mild to moderate disease progression.
4. There is severe disability, but the patient is still able to walk or stand unassisted.
5. Patient needs a wheelchair or is bedridden unless assisted.

Accordingly, this proposed schema foundation provides a weighted scoring approach that allows for an emphasis to still be placed on motor functionality. This is depicted in Figure 3 in the separation of motor function into the subcategories of fine motor (eg, involving the movement of the small muscle groups in the hands, fingers, and wrists), gross motor (eg, involving the movement of large muscle groups for functional mobility such as gait), balance (eg, assessing the person’s ability to both statically and dynamically distribute weight evenly, enabling them to remain steady), and reflexes (eg, involving a reactionary motor response to an outside stimulus, which can be tactile, visual, or aural). This proposed schema foundation would maintain functionality for the representation of unilateral, bilateral, and balance deficits in the lowering of fine motor, gross motor, and balance scores while possibly also allowing for greater discernment between the different types of motor symptom effects (eg, tremor vs rigidity). Furthermore, this foundation takes inspiration from the MDS-UPDRS [14] to include the assessment of all remaining functional areas of neurocognition (eg, memory, speech, language, executive function, autonomic function, behavior, sleep, and sensory function) via the use of objective digital biomarkers and features from digital health technology (eg, smartphones, tablets, and wearable devices), PROs, and clinician observations, all of which are essential in the accurate staging of this neurodegenerative disease.

This proposed system outlines a schema foundation that is intended to be expanded upon with further insights from clinicians, patients, and researchers. The main intent of this schema foundation is to emphasize the inclusion of objective features related to all functional areas of neurocognition. However, given the prevalence and expansion of digital health technology in the neurocognitive assessment space, the continued collection of novel health-related data (eg, digital biomarkers and PROs), and the implementation of various ML approaches (eg, neural networks, random forests, support vector machines, and transformers) for the interpretation of collected features, alongside clinician expertise, data-driven updates to this proposed system would ultimately be both necessary and proper. Subsequently, via the use of these ML approaches, deeper analysis would be possible for understanding comorbidities, treatment effects, and condition prognosis. Further improvements, yielded from ML techniques and relating to feature importance, would also include the updated weighting of features (eg, digital biomarkers, clinician observations, and PROs), allocation and reallocation of points between different functional areas, the inclusion of additional relevant subfunctional areas of neurocognition (eg, subcategories of long- vs short-term memory), and changes to the cutoff score threshold values, among others.

## Discussion

### Overview

Personalized medicine should always be a prioritized goal in health care, and it is necessary to optimize this care for an individual’s quality of life [118]. To achieve this type of personalized medicine for individuals with PD, clinicians need further knowledge on specific patient characteristics across all functional areas of neurocognition to develop personalized pharmacological and interventional protocols in an evidenced-based manner [119]. However, current clinical assessment protocols for the staging of PD use predominantly motor functionality, and thus many neurocognitive functional areas (eg, memory, speech, language, executive function, behavior, autonomic function, sensory function, and sleep) are overlooked. Although the cardinal symptoms of PD are predominantly motor based (eg, bradykinesia, rigidity, tremor, and postural instability) [120], it is now well recognized that PD is far more than just a motor-deficit disorder (49). Nonmotor symptoms have a significant impact on quality of life (perhaps more so than motor symptoms), and they have prognostic relevance with psychosis and dementia driving the need for nursing home placement [121,122]. This is further addressed by the extended literature review results in Table 2, which show that >62% (74/119, 62.2%) of the articles have a focus on nonmotor function being assessed using digital health technology.

Given the proposed schema and the further integration of digital health technology, expansive efforts as well as the impacts of personalized medicine should be considered across different clinical areas (eg, neurology, pharmacology, physical therapy, speech-language pathology, and occupational therapy). This includes use in disease classification, stage prediction, and the possible categorization of different PD subtypes (similar to amyotrophic lateral sclerosis [ALS], in which both limb and bulbar presentations of the disease occur [123]) [41,45,124] or to rule out PD mimics (eg, atypical parkinsonisms such as multiple system atrophy, progressive supranuclear palsy, and dementia with Lewy bodies). As personalized medicine prioritization is necessary for advancing care for individuals with PD, the objective assessment of each neurocognitive function (eg, motor, memory, speech, language, executive function, autonomic function, sensory function, behavior, and sleep) is imperative in guiding and assessing each point of care across clinical contexts while also providing patients with improved clinical outcomes. As such, these clinical impacts are directly related to the use of AI and ML to provide insights that may supplement clinical expertise while also driving predictive and personalized medicine–based approaches.

### Neurology

As PD is one of the world’s fastest-growing neurological disorders, where access to neurological care is a rare privilege for millions of people worldwide, digital health technology provides accessibility and continuous monitoring capabilities [5,125,126]. Remote monitoring, using these technologies, combined with telemedicine capabilities, can close the accessibility gap. Currently, the standard of diagnosis and staging requires in-person clinic visits where an expert (eg, neurologist or movement disorder specialist) assesses the disease symptoms while observing the patients as they perform a series of clinical assessments. However, given the time constraints of standard clinical visits (eg, every 3-6 mo) in conjunction with the nature of PD symptoms (eg, with varying intensity and timing), clinicians, typically, are unable to adequately discuss specific or personal issues with the patient and caregiver regarding the disease [2,127]. Therefore, the continued use of outdated assessment protocols (eg, H&Y and MDS-UPDRS) that are strictly limited to a subset of functional modalities and the subjective reporting of outcomes, leaves much unknown in the presentation, progression, and treatment of PD [128,129]. Therefore, digital health technology is intended to augment this process for neurologists and movement disorder specialists by providing objective measures across all functional areas of neurocognition in a longitudinal manner [2] to enable them to make comprehensively informed decisions regarding interventional therapies (eg, pharmacological treatment, physical therapy, speech-language pathology, and occupational therapy), while also allowing for the needs of the patient to be addressed. Similarly, these objective measures would benefit patients and researchers in the clinical study setting by facilitating the detection and monitoring of the effect of therapeutics on motor and nonmotor symptom severity and progression over time.

### Pharmacology

Due to the heterogeneity of clinical interactions, complications in diagnosis and staging may persist because motor and nonmotor symptom fluctuations can be influenced by medication timing, variability in efficacy, and differences in the duration of effect relative to the timing of the assessment. As the symptoms presented during clinic appointments over time may not reveal all issues that are present at home, and there may be substantial variations between the peak (eg, the highest level of a medication’s concentration in the blood) and trough (eg, the lowest level of a medication’s concentration in the blood) states of pharmacological interventions, it is also challenging to prescribe the right dose of medication and schedule doses to prevent trough states [129]. However, sudden fluctuations in peak and trough states may be recognizable with wearable sensors that have automated algorithms [130]. To handle the challenge of recognizing these peak and trough fluctuations with respect to the symptom-specific functional areas of neurocognition, remote at-home monitoring would allow health care professionals to better prescribe medications, and in the event of more complicated cases, it would help them to decide the optimal time frame for advanced, more invasive PD treatments (eg, deep brain stimulation or intraduodenal levodopa infusion) [46,131].

### Physical Therapy

The adoption of digital health technology by physical therapists to address current, evolving, and future health care needs has been increasingly advocated for more than a decade. Notably, in 2009, the American Physical Therapy Association sponsored the Physical Therapy and Society Summit at which participants proposed that the technological drivers of change in physical therapist practice should include activity monitoring and telerehabilitation efforts [47]. Although physical therapists typically tailor exercise interventions to meet the needs of a given patient, there is little evidence to guide these decisions [132]. Given the vast variability in disease characteristics and subsequent functional consequences among persons with PD, a precision medicine approach is needed to optimize personalized exercise programs tailored to their functional abilities [48]. This is further advocated in an expansion of previously presented work where neurocognitive functional improvements across both motor and nonmotor symptom-specific functionalities (eg, motor, memory, speech, and executive function) were mapped in relation to specific physical intervention programs (eg, aerobic activity, noncontact boxing, functional strength, and yoga) across PD stages [8]. Although there is a continued research need in the mapping of these physical interventions to symptom-specific improvements, it is imperative to modernize PD assessment protocols is imperative to drive these personalized interventions. Furthermore, the use of digital health technology could then be expanded to allow access to a home-based approach to therapeutic activity and extend the benefits of in-person training with physical therapists [7].

### Speech-Language Pathology

As nearly 90% of people with PD develop speech disorders, speech assessments have a crucial role in the clinical diagnosis and monitoring of PD [133,134]. In many cases, speech disorders—specifically hypokinetic dysarthria (characterized by a breathy voice, an increased rate of speech, monopitch, reduced syllable stress, and imprecise articulation)—are the first indications of PD. Furthermore, in the later stages of PD, changes in speech (ie, increased spasticity characterized by harsh-strangled voice features) usually indicate more cognitive involvement as seen in certain types of dementia. In addition, dysphagia (ie, swallowing disorders manifested by delayed swallow response, slow tongue movements, and drooling—all due to increased rigidity) is a common occurrence in PD, affecting an estimated 40% to 90% of individuals [135]. The ability to effectively collect and objectively assess neurocognitive and oral motor features associated with speech (eg, pitch, loudness, articulation, voice quality, respiration, resonance, and prosody) through the use of digital health technology would enable both precise assessment measurements for the diagnosis of individuals with PD and the monitoring of the therapeutic benefits of effective and targeted treatment of the presented symptoms [49,134].

### Occupational Therapy

Digital health technology and telemedicine-based approaches for occupational therapy are also necessary in the provision of safe and effective therapeutics. Although physical activity, specifically exercise, can improve motor and nonmotor function and quality of life in people with PD, these benefits are likely derived only if the person engages in adequate levels of physical activity in daily life. Therefore, occupational therapists have a role in helping people with PD incorporate and maintain physical activity in their daily routine (activities of daily living) as an aspect of health management and maintenance [138]. With the implementation of mobile technology in this space, the continued development of therapy programs and neurocognitive assessment methods will allow for usable, safe, low-cost, and accessible assessments for all [50].

### Disease Classification

The pervasiveness of digital devices and the emergence of digital health technologies are helping to address the problems of early PD detection and risk prediction even in a nonclinical environment [11]. These devices allow for the accurate characterization and recognition of discriminating features via the use of ML algorithms and AI-enabled models for detection and assessment [38,51,136]. Furthermore, the use of this information allows for the reevaluation and standardization of clinical scale updates, which can ultimately allow for novel digital health systems and disease monitoring [139]. Especially in the early stages, parsing out PD from atypical parkinsonism syndromes such as progressive supranuclear palsy or multiple system atrophy can be difficult, and the standard clinical evaluation can lead to misdiagnosis. Diagnostic accuracy should be enhanced with the use of digital health technology supplemented by ML and AI to differentiate PD from atypical PD with implications regarding prognostic discussions and clinical trial enrollment. As the prognostication of PD is an aim of high importance, updates regarding the collection of objective data, the modeling for the characterization and recognition of discriminating features, and the updated standardization of clinical scales using digital health technology are necessary to drive this pivotal aim.

### Stage Prediction

ML has become increasingly significant in health care for stage and outcome prediction [9]. These approaches have penetrated the neurology and digital health technology crossover space to address challenges regarding neurodegenerative movement disorders such as PD [140,146]. Prognostication in PD, regardless of the disease state, is important to patients and difficult for clinicians. Predicting how the disease will evolve and when patients will reach clinical milestones is a major scientific challenge. The application of digital health technology can add objective motor and nonmotor symptom data to these calculations; for example, gait patterns consistent with the freezing of gait and labile blood pressure in the setting of neurogenic orthostatic hypotension, detected subclinically through ML, may predict falls or the development of dementia, both important milestones in PD progression. Monitoring neurocognition over time in a large cohort of patients with PD would allow for retrospective analysis of the recorded data compared with the attainment of disease milestones to help create predictive models. Predicting the time period in which a patient progresses to a more advanced disease stage (eg, from stage 1 to stage 2 or higher) is highly necessary for clinicians in making medical decisions (eg, in terms of treatment and intervention options). To go further, data collection procedures should be defined to adequately collect data for these patients as they progress through disease stages over time. This would completely revolutionize PD care across all types of therapeutics and interventions because clinicians could inform patients about their individualized likelihood of having critical disabilities and assistive-device needs, helping them better prepare for the future [141]. The inclusion of stage prediction still requires additional work; however, this component will provide substantial benefits for personalized treatment protocols once it is completed.

### AI and ML for the Categorization of PD Based on Presented Symptoms

With the continued adoption of digital health technology specific for PD, it may be possible that objective assessment features allow for better classification of PD. The use of AI and ML techniques such as k-nearest neighbors, random forests, support vector machines, neural networks, and other modeling methods can allow for improved accuracy compared to current staging methods [116,142]. These models may also be readily used to better assess subtype manifestations of the condition at either the prodromal stage or across novel PD phenotypes (similar to ALS, in which both limb and bulbar presentations of the disease occur [52,123]). The use of AI and ML for novel unsupervised learning pathways (eg, principal component analysis, t-distributed stochastic neighbor embedding, and uniform manifold approximation projections) [143,144] could completely revolutionize PD care, given the novel insights that could allow for deeper understanding related to pharmacological and therapeutic interventions, prognosis, and comorbidity manifestation likelihood, among others [8]. Given the nature of this “designer disease, ” these differentiated symptom classifications would be imperative for the formation and provision of personalized care across all clinical settings.

### Summary of Clinical Impacts

**Figure 4:**
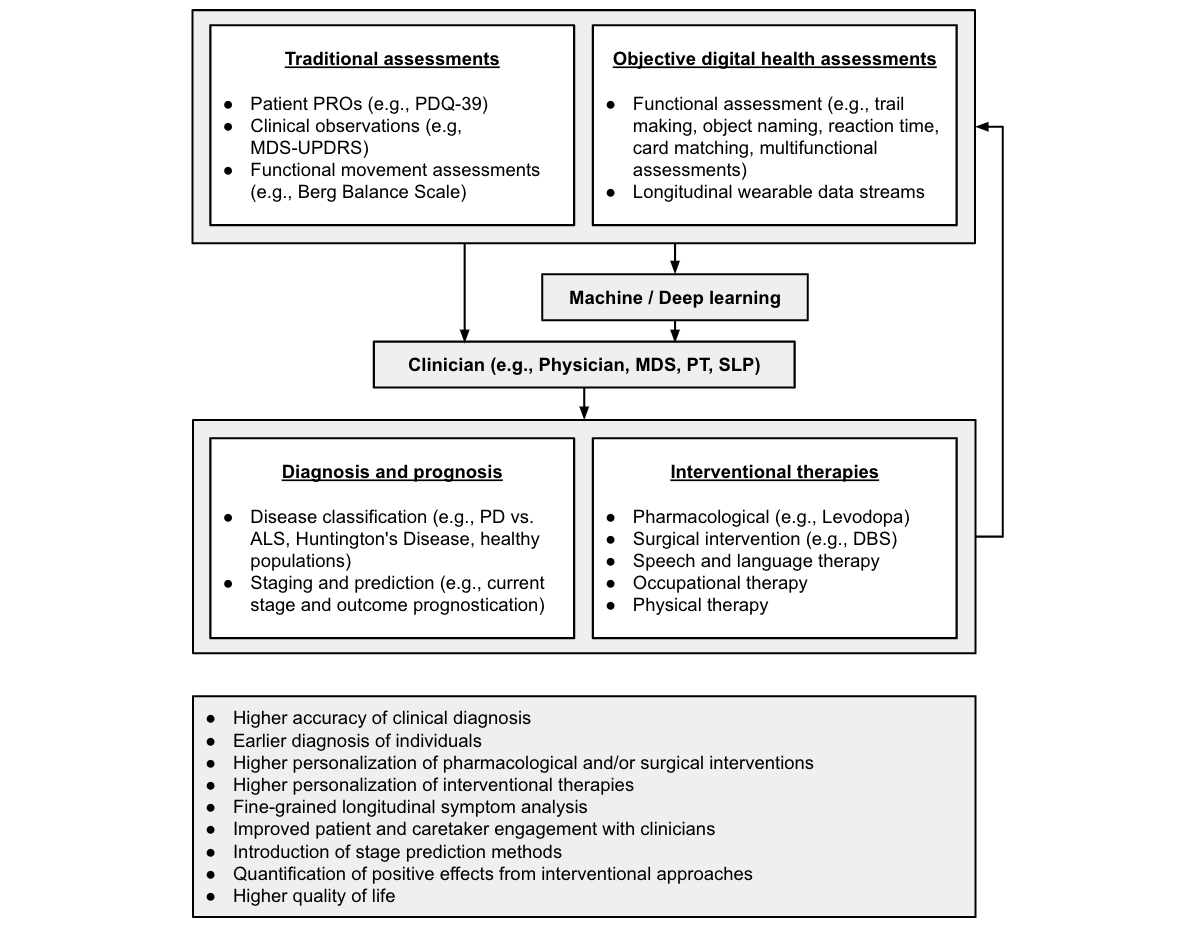
Benefits of integrating digital health technology into disease classification, staging, and prediction. ALS: amyotrophic lateral sclerosis; DBS: deep brain stimulation; MDS-UPDRS: Movement Disorder Society–Unified Parkinson’s Disease Rating Scale; PD: Parkinson disease; PDQ-39: Parkinson’s Disease Questionnaire-39; PRO: patient-reported outcome.

Given the many discussed impacts of digital health technology, Figure 4 depicts a visual flow-based representation of new staging systems. As this technology provides the ability to collect objective data from digital health tools (eg, smartphones and wearable devices) and analyze the data through ML and deep learning techniques (eg, random forests, support vector machines, and neural networks), given expansive data inputs from traditional and objective digital assessments (eg, PROs, clinical observations, functional movement assessments, digital health assessments, and longitudinal wearable data streams) at multiple influential events (eg, semiannual assessments with clinicians and with respect to pharmacological interventions and other therapies), clinicians are afforded critical information to make more informed decisions for personalized diagnosis, staging, and intervention recommendation protocols. Although future work will necessitate more robust data collection efforts and the formalization of ML models for outcome prognostication and therapeutic recommendation, the application of digital health technology in this capacity has already provided clinicians with valuable objective features necessary for the objective assessment, accurate classification and staging, and longitudinal monitoring of PD (Tables S1-S3 in Multimedia Appendix 1). Subsequently, the increased and continual integration of this digital health technology sets a foundation that can meaningfully process this wealth of data from multiple sources while enabling neurologists and movement disorder specialists to provide personalized intervention protocols, adequately discuss and address specific personal issues with the patient or caregiver regarding the disease and its progression at the time of clinical visits, and ultimately allow individuals living with PD to experience a better quality of life [137].

Finally, with the modernization of neurocognitive monitoring using mobile technology, it is noted that this concept can and should be applied to other neurodegenerative diseases (eg, Huntington disease, ALS, Alzheimer disease, multiple sclerosis, and other types of dementia) as each of them present with progressive degeneration that may involve both neurological and cognitive deficits [145]). Furthermore, this concept could naturally be extended for a deeper understanding of the confounding effects of comorbid conditions as well as nuanced responses to multiple interventional programs. Using digital health technology for objective symptom-specific assessments should be advocated as a supplement to functional movement assessments, PROs, and clinician observations in the provision of personalized and optimized care for individuals across all neurodegenerative conditions.

### Limitations and Future Directions

While the proposal to modernize clinical staging of PD using digital health technology builds on established scales such as the H&Y and MDS-UPDRS, it also seeks to enhance precision and functionality by integrating objective, symptom-specific assessments through advanced device capabilities [2]. However, this integration also presents several challenges that need to be addressed. One main limitation revolves around privacy concerns regarding the collection, storage, and sharing of sensitive patient data. As this proposed staging mechanism is intended to be usable across a variety of clinical settings (eg, neurology, pharmacology, physical therapy, speech-language pathology, and occupational therapy), the process of collecting, storing, and sharing data requires significant foresight for regulatory compliance as well as clear consent processes to protect patient information [53]. In addition, as these devices allow for the collection of large amounts of data of various types (eg, audio, video, and wearable sensor stream data), the storage of these data becomes a concern for both longitudinal analysis and system scalability [54]. Next, accessibility presents additional challenges, particularly in resource-limited settings or among older people who may struggle with technological literacy or lack access to advanced digital tools [55]. The development of affordable and user-friendly systems that can function across diverse health care environments is essential to ensuring equitable implementation. While digital health technology (eg, smartphones, tablets, and wearable devices) is becoming more readily available in the health monitoring space, limitations remain in ensuring widespread access for all populations [147]. As such, any inability to assess specific functional areas of neurocognition due to a lack of digital health technology devices would still cause reliance on subjective assessment and PROs. Furthermore, developing an ideal system for assessing each functional area of neurocognition is reliant on the formation of robust data collections that can yield optimal feature sets across each area of neurocognition. While Table 3 highlights specific manuscripts that yield objective features across each functional area of neurocognition, currently there is no dataset that contains data from all these devices across all areas. Even with the use of currently available clinical databases that contain objective data from many key neurocognitive functions, in addition to ongoing data collections with populations of people with PD, further specifications are also necessary for development regarding specific features of importance. Given the rapid pace of advancements in ML and wearable technology, there is also the need for periodic updates within the proposed system; however, it is believed that with the digitization of current assessment systems paired with a proposed schema that is based on current clinical gold standards, these challenges can be addressed through collaborative and iterative approaches that will translate this proposed framework into a practical, widely applicable tool for staging and managing PD.

### Conclusions

Digital devices, including smartphones, tablets, and wearables, provide the capability to obtain accurate, objective measures and PROs for neurocognitive monitoring; allow for a much more fine-grained approach to longitudinal monitoring across all functional areas of neurocognition; and lead to the enhancement of personalized rehabilitative efforts, ultimately yielding higher patient quality of life. The creation, implementation, and continued evolution of mobile device technology for the continuous assessment of individuals with neurodegenerative diseases, including PD, should be used to transform the process of clinical staging into a much more comprehensive and personalized monitoring approach. This newly proposed foundation would ultimately provide benefits to all parties involved (eg, neurologists, clinicians, researchers, patients, and caregivers); yield a basis to expand upon, given future work in the field; and is intended to aid in the transformation of the way neurodegenerative diseases are understood, studied, and cared for.

## Appendix

Multimedia Appendix 1 Highlights from the extended literature review of manuscripts advocating for the use of digital health technology in the modernization of the assessment, classification, staging, and longitudinal monitoring of Parkinson disease.

## Abbreviations

AI: artificial intelligence
ALS: amyotrophic lateral sclerosis
H&Y: Hoehn and Yahr Scale
MDS-UPDRS: Movement Disorder Society–Unified Parkinson’s Disease Rating Scale
ML: machine learning
PD: Parkinson disease
PRO: patient-reported outcome
