# Supplementary material for: Modernizing the Staging of Parkinson Disease Using Digital Health Technology
Source: J Med Internet Res. 2025 Apr 4;27:e63105. doi: 10.2196/63105 (PMC12008700; doi:10.2196/63105)
Supplement: Multimedia Appendix 1 [file jmir_v27i1e63105_app1.docx]

## Multimedia Appendix 1

Highlights from systematically reviewed manuscripts advocating for the use of digital health technology in the modernization of the assessment, classification, staging, and longitudinal monitoring of Parkinson’s Disease.

Table 5. Highlights from systematically reviewed manuscripts advocating for modernizing the assessment of PD via digital health technology for *both motor and non-motor* neurocognitive functions.

| Citation | Excerpt(s) on the use of Digital Health Technology for Neurocognitive Assessment & Care of Individuals with PD |
| --- | --- |
| Espay et al. (40) | “The time has come to further develop and integrate mobile health technologies into the routine assessment and care of patients with PD” AND “This collaborative endeavor will encourage the development of integrated, multichannel systems that can achieve more sophisticated characterization of patients’ function, better tailoring of symptomatic therapy, greater patient engagement and self-assessment by patients, and overall improved health care outcomes.” |
| Ellis et al. (7) | “Digital therapeutics need not be limited to approaches utilizing exercise or to those aimed to address motor aspects of the disease. Non-motor aspects of PD can also be addressed effectively via digital means.” AND “Advances in artificial intelligence coupled with the central role of mobile technology in everyday life has expanded the application of digital therapeutics to healthcare.” |
| Sacks et al. (88) | Digital health technology offers the potential for objective measurement of tremor, gait deficits, freezing of gait, postural instability, upper limb motion, leg agility, rigidity, and motor fluctuations in addition to abnormalities in cadence, tonal variation and fluency of speech in PD patients. |
| Hansen et al. (25) | Care of patients with PD will dramatically change in the upcoming years where the nationwide implementations of patient-controlled electronic health records and technology-based home monitoring systems will be the cornerstones of this revolution. |
| Elm et al. (89) | As PD patients are evaluated by their treating clinician for in- person visits infrequently and symptom manifestations tend to be fluctuating, digital health technology to capture the objective data in a more ecologically valid setting would be helpful for clinicians to inform therapeutic decisions. |
| Erb et al. (90) | Digital measurement tools including mobile and wearable technologies have been widely acknowledged as promising for improving upon the remote monitoring of people with PD. |
| Far et al. (91) | Digital biomarkers being collected frequently over a long period of time can provide an objective, ecologically valid, and more detailed understanding of the inter- and intra-individual variability in disease manifestation in daily life. |
| Morgan et al. (43) | There is recognition voiced by the International Parkinson and Movement Disorders Society Task Force on Technology, that technology could provide a rich source of objective granular data which captures the disease and treatment-related fluctuations of this complex condition via technological sensors. |
| Ganesan et al. (71) | Researchers should develop online therapy programs, and online-based physical and cognitive assessment methods, which should be usable, safe, low cost, and accessible for all. |
| van Uem et al. (92) | The revolutionary development of using mobile devices in domestic environments to objectively measure personal functioning opens entirely new assessment strategies in chronic disorders such as PD. |
| Dorsey (93) | Digital health technology is poised to “transform major, real-world sectors” including health care. |
| Marquis-Gravel et al. (94) | Technological advances that leverage electronic health record data sources, mobile devices, digital mHealth applications, biosensors and wearables, web-based platforms, and AI/ machine learning are strongly supported by the FDA to guide regulatory decision making, and are expected to improve the cost-effectiveness and diversity of clinical trials. |
| Vizcarra et al. (95) | Highly dynamic and user-friendly technological advances of recent years enable the development and validation of an accepted trackers that simultaneously assesses motor and non-motor function. |
| Bouça-Machado et al. (96) | Mobile health technologies, which can collect and connect clinical and non-clinical information to feed the existing health informatics systems, seem a valuable solution to address the growing challenges of cost and provision of quality healthcare. |
| Del Din et al. (97) | Mobile technology are revolutionizing approaches to healthcare due to their utility, accessibility and affordability and they are positioned to transform PD management through provision of individualized, comprehensive, and representative data. |
| Zhang et al. (11) | The pervasiveness of mobile devices and the emergence of mobile health technologies are addressing the problems of early PD detection and risk prediction even in a non-clinical environment. |
| Schneider et al. (86) | The expanding power and accessibility of personal mobile technology provides an opportunity to reduce burdens and costs of traditional clinical site-centric therapeutics in PD while generating novel and objective insights. |
| Chandrabhatla et al. (42) | In the future, a transition to truly continuous PD symptom monitoring has the greatest potential by leveraging easy-to-use mobile applications on smart devices that integrate quantitative and qualitative data. |
| Lim et al. (98) | Precision medicine is advancing rapidly, and it is likely that technologies such as smartphone and wearable devices will be increasingly used to supplement clinical assessments. |
| Dorsey et al. (99) | Health improvements will likely come from changes in behavior, improved data on which to base clinical decisions, and eventually, greater access to care - likely through the same devices that individuals are using to advance research. |
| Perry et al. (100) | It has been demonstrated that a vast amount of effort that has gone into the development and refinement of mobile outcome assessments for PD and the wealth of scientific evidence supports the use and application of mobile technologies in future PD clinical research studies. |
| Templeton et al. (8) | With the collection of patient reported outcomes, clinician observations, and objective digital features from mobile-based assessment tools, clinicians can accurately assess symptom- specific functional deficits across different areas of neurocognition and subsequently provide data-derived intervention protocol recommendations. |
| Kim et al. (101) | Customized mobile apps have beneficial impacts on adherence to exercise as well as physical activity, depression, and quality of life in patients with PD. |
| Sun et al. (102) | “Swift advancements in digital health technologies enable the  continuous collection of health data in natural settings, and the  enhancement and transparency of algorithms further catalyze the  clinical integration of digital biomarkers.” |
| Hu et al. (103) | Many PD patients have positive attitudes toward PD-related smartphone apps and the increasing smartphone usage for monitoring PD is a promising strategy disease management. |
| Lauraitis et al. (73) | Classification models cover a very wide range of possible symptoms which implies high potential for the determination of the deteriorating status of each subject’s health state for early screening and disease progress monitoring. |
| Carter et al. (53) | Mobile devices allow neurologists and other treating clinicians access to real-time measures of disease progression and the impact of medication over periods not previously possible. |
| Gatsios et al. (104) | Digital health technology, are considered in PD to provide valuable ecological data between face-to-face visits and improve monitoring of motor symptoms remotely. |
| Artusi et al. (105) | There is an increased interest in the use digital health technology to replace clinical scales for monitoring the disease course and assessing the therapeutic effect of patients with movement disorders. |
| Lee et al. (65) | Mobile health devices have enabled improvements in diagnosis and treatment, as well as connection with distant patients. |
| Xu et al. (106) | Innovations in remote assessment technology are highly feasible for their transforming power in the clinical management of PD deep brain stimulation and research while increasing safety and being a well accepted tool. |
| Alberts et al. (107) | Moving beyond subjective visits in the treatment of neurological disease is a critical step in propelling telemedicine to a preferred method of medical visits. |
| Timotijevic et al. (84) | Mobile health based clinical decision support systems should be implemented to provide appropriate and reliable evidence to aid clinical judgments and reduce the uncertainty inherent in natural decision-making contexts experienced by clinicians. |
| Kenny et al. (108) | In the design and implementation of crucial technology for PD, improved user engagement and the adoption of and compliance with wearable devices, can lead to more accurate disease- and self-management. |
| Lauraitis et al. (109) | The fusion of computational intelligence with the advancement of mobile devices and their embedded sensors can serve as an efficient support in automation for medical diagnosis. |
| Prince et al. (110) | Due to the subjectivity of current clinical assessment systems, the use of digital health technology to identify digital biomarkers that are capable of objective disease quantification are now widely being sought. |
| Goñi et al. (111) | Smartphone-based digital biomarker assessments provide objective measures of daily-life tasks and thus hold the promise to improve diagnosis and monitoring of PD. |
| Mughal et al. (112) | The lack of resources available to provide individualized face-to- face medical interactions has led to rapid technological advancements in this field that allow for remote monitoring and the precise and timely management of neurodegenerative diseases like PD. |
| Adams et al. (113) | A commercially available smartwatch and a smartphone research application captured key motor and non-motor features of early, untreated PD" AND "This multicenter study in early, untreated PD provides valuable data on multiple digital measures derived from widely available devices. |
| Mammen et al. (114) | The findings presented here contribute to understanding the relevance, risks, and benefits of digital measures to monitor symptoms of early PD from the participant perspective. |

Table 6. Highlights from systematically reviewed manuscripts advocating for modernizing the assessment of PD via digital health technology for *motor specific* neurocognitive functions.

| Citation | Excerpt(s) on the use of Digital Health Technology for Neurocognitive Assessment & Care of Individuals with PD |
| --- | --- |
| Zhan et al. (115) | A smartphone-derived severity score for Parkinson’s Disease is feasible and provides objective measures of motor symptoms inside and outside the clinic that could be valuable for clinical care and therapeutic development. |
| Jette et al. (63) | Information technology must play a central role in the redesign of the health care system if a substantial improvement in quality care is to be achieved. |
| Miller Koop et al. (116) | Mobile device platforms can provide clinicians with a portable, accurate, simple-to-use system that can objectively and immediately quantify postural and gait stability with reliable biomechanical measures. |
| Lipsmeier et al. (117) | Digital health technology enables remote and therefore frequent measurement of motor signs to provide more robust, reliable, and valid-quantification of disease severity and its changes over time. |
| Billnitzer et al. (118) | As it is well recognized that telemedicine and remote medicine will continue to become more common place, it is important to encourage high quality home videos as a supplemental tool in diagnosis and management of patients with movement disorders. |
| Sisti et al. (119) | Adopting a big data approach for analyzing movement disorders may facilitate both our understanding of disease progression and help guide clinical decision-making during the treatment of these conditions. |
| Rupprechter et al. (120) | Quantitative analysis of gait examination has not yet been widely adopted for use by clinicians assessing patients despite readily available technologies enabling this for decades. |
| Lieber et al. (121) | There is a great need for low-cost technology-based systems able to provide objective measurement of a patient’s movement in hospital, clinic, and longitudinal settings. |
| de Carvalho Lana et al. (122) | Smartphone technology and activity monitors can be used to assess physical activity for individuals with PD, and allows for assessment and intervention purposes. |
| Arroyo-Gallego et al. (123) | Current practices not only lack better monitoring of PD progress, but also do not provide consistent and objective evaluation of the measured signs and symptoms. |
| Corrà et al. (124) | Wearable health technology for the assessment of PD yields many advantages compared to clinical scales and conventional lab-based tools, as it allows for more consistent/reliable results. |
| Yang et al. (49) | It is necessary to develop an objective long-term assessment system of motor function in PD patients, with characteristics of sensitivity, accuracy, portability, and objectivity via the evolution of mobile and computer technology. |
| Pan et al. (125) | With the rapid development of sensor technology, cloud computing, and ubiquitous access to the Internet from mobile devices, digital health technology has spurred the development assessment systems that can extend PD monitoring from intermittent clinic-based approaches by leveraging current mobile device and powerful cloud computing. |
| Cohen et al. (126) | Alternative tools for continuous and objective monitoring of PD motor symptoms are needed to complement clinical assessments and patient reported outcomes. |
| Chang et al. (127) | We need digital health technologies that can monitor for clinical onset of disease to help determine when to initiate expensive and potentially invasive therapies. |
| Jakob et al. (128) | Mobile systems have the potential to record gait patterns over several hours in flexible environments, including a patient’s daily life which is a major benefit for physicians as clinical diagnostics are limited to a short timeframe in the hospital during the doctor’s visit. |
| Surangsrirat et al. (129) | Over the last few years studies on objective measures from mobile devices have been introduced and investigated with the aim to quantify disease severity based on statistical data without the bias of the specialist or patient. |
| Iakovakis et al. (130) | There is an increasing need for quantitative assessment of PD using widely accessible Information and Communication Technology that can assist the screening of PD high-risk population and remote monitoring of the PD symptoms. |
| Ruokolainen et al. (131) | Parkinson’s Disease symptoms’ clinical assessment suffers from significant interrater variability due to its subjective nature; therefore, an objective assessment method based on automatic analysis of movement data recorded continuously would have the potential to significantly improve the effectiveness of care for patients with PD and other neurological diseases. |
| Jauhiainen et al. (62) | To handle this challenge of recognizing fluctuations of symptoms of varying intensity and timing, remote monitoring at home would help health care professionals prescribe medication and aid in deciding the optimal timeframe for secondary, invasive PD treatments such as deep brain stimulators or intraduodenal levodopa infusions. |
| Dai et al. (132) | Together with the assessments of bradykinesia, rigidity, and the side effects of medication/surgical treatments such as dyskinesia, paresthesia, and other non-motor symptoms, more features should be evaluated in a single system for the overall assessment of PD. |
| Vila-Viçosa et al. (133) | “It is apparent that there is a necessity to measure relevant mobility outcomes in PD patients continuously and objectively to provide more personalized and ecological perspectives on patients’ real-world performance.” |
| Rehman et al. (79) | It is hoped that presented results are a step towards the adoption of comprehensive mobile-based approaches to find the best movement features at the early stages of PD which may be applicable for better classification at the prodromal stage or even between PD phenotypes. |
| Ferrari et al. (134) | Mobile devices pave the way for a new generation in monitoring and intervention. |
| Azami et al. (135) | Mobile phone-based approaches will become even more important to having widely available and inexpensive screening tools for early detection of disease to enable treatment early in the disease course. |
| Debelle et al. (44) | Assessing self-reported medication adherence, tracking motor complications, and monitoring mobility in people with mild-to-moderate Parkinson’s disease are feasible using this novel DHTS and a motor complications diary. |
| Purk et al. (136) | There is potential in applying device-based tools that utilize sensors and patient reported outcomes for surveillance and diagnostics of movement disorders. |

Table 7. Highlights from systematically reviewed manuscripts advocating for modernizing the assessment of PD via digital health technology for *non-motor* *specific* neurocognitive functions.

| Citation | Excerpt(s) on the use of Digital Health Technology for Neurocognitive Assessment & Care of Individuals with PD |
| --- | --- |
| van Wamelen et al. (137) | Following the increased availability and use of wearable technology in PD, the focus of these technologies should also shift towards the broad non-motor symptoms of PD. |
| Zhang et al. (138) | In the context of smartphone apps for PD self-management, patients are eager to gain access to education, record motor and non-motor symptoms to better self-manage symptoms, and communicate with doctors online via mobile devices. |
| Bérubé et al. (139) | “As the health care system is currently unfit to sustain the prevention and management of chronic and mental health conditions while containing its costs, continuous and personalized smartphone-based interventions have been developed to provide scaled-up behavioral support.” |
| Singh et al. (140) | Smartphones are the most ubiquitous mobile device, and their abilities, cost-effectiveness, and objective measures compared to traditional clinical methods, could revolutionize the industry. |
| Duffy et al. (85) | Mobile technology can offer a range of opportunities to support people with PD through structuring activities, adding gamified elements to increase enjoyment, and providing positive reinforcement and feedback. |
| Rosenblum et al. (141) | Smartphones enable “healthcare in the pocket” and are becoming an increasingly important platform for delivering health interventions as they offer a valid, feasible, and acceptable method for collecting health-related data. |
| Laganas et al. (4) | “The development of accessible, technology-based methods for longitudinal PD symptoms tracking in daily living, offers the potential for transforming disease assessment and accelerating diagnosis.” |
| Tsanas et al. (142) | It is demonstrated that that mobile phone technology could be useful in telemonitoring PD symptom severity, further endorsing previous findings that speech may offer a convenient framework for remote assessment. |
| Arora et al. (143) | “The research community has embraced the use of technology in the hope of facilitating objective, sensor-based PD assessments.” |
| Palacios-Alonso et al. (144) | “The ongoing assistance of patients with chronic neurodegenerative disorders demands fast response solutions, which can be facilitated by Information and Communication Technologies.” |
| Lauraitis et al. (69) | As speech disorders in PD tend to progress over time, classification methods can function as a decision support system for monitoring the health state of patients and provide insight about disease status. |
| Domenici et al. (145) | Preemptive screening using mobile devices is crucial to investigating the clinical condition itself and counteracting the deterioration of the patient’s quality of life. |
| Janssen Daalen et al. (146) | Current gold-standard scales such as the MDS-UPDRS come with several challenges as they require substantial assessment time, are conducted episodically and are based on subjective interpretation, which may lead to considerable measurement errors over time. |
| Schalkamp et al. (147) | Digital sensors enable a passive and continuous collection of information in the home environment compared to in-clinic visits that use self-report and clinical rating scales that pose limitations such as the time and cost of in-person visits, subjectivity of self-report, and inter-rater variability. |
